# CAR-exosomes derived from immune cells: an emerging nanoscale vanguard in overcoming tumor immunotherapy hurdles

**DOI:** 10.3389/fimmu.2025.1655095

**Published:** 2025-08-19

**Authors:** Xiaoyan Zhao, Bin Zhao, Yan Sun, Aiguo Liu

**Affiliations:** ^1^ Department of Pediatrics, Tongji Hospital, Tongji Medical College, Huazhong University of Science and Technology, Wuhan, China; ^2^ Shanxi Province Key Laboratory of Oral Diseases Prevention and New Materials, Shanxi Medical University School and Hospital of Stomatology, Taiyuan, China; ^3^ Department of Thoracic Surgery, Union Hospital, Tongji Medical College, Huazhong University of Science and Technology, Wuhan, China; ^4^ Department of Pediatrics, Tongji Hospital, Tongji Medical College and State Key Laboratory for Diagnosis and Treatment of Severe Zoonotic Infectious Disease, Huazhong University of Science and Technology, Wuhan, China

**Keywords:** CAR, exosomes, cancer, immunotherapy, cell-free

## Abstract

Chimeric Antigen Receptor (CAR)-engineered cell therapies excel against hematologic malignancies, however, their efficacy in solid tumors is hampered by toxicity, poor tumor infiltration, immunosuppressive microenvironments, limited persistence, and expansion difficulties. Recently, exosomes derived from CAR-immune cells (CAR-Exosomes) have emerged rapidly as an innovative therapeutic platform. CAR-Exosomes, utilizing nanoscale communication pathways, inherit their parental cells’ tumor-targeting capabilities while offering distinct advantage. These advantages encompass low immunogenicity, enhanced tissue penetration, and versatile drug-loading capacity, presenting a promising approach to circumvent the limitations of traditional cell therapies. This review systematically summarizes the core challenges for CAR-T, CAR-NK, and CAR-M cell therapies and emphasizes recent advancements in CAR-Exosomes, including their molecular characteristics, targeted recognition mechanisms, tumor-killing pathways, biosafety, and engineering strategies. Furthermore, it also discusses the key challenges and strategies in the clinical translation of CAR-Exosomes. In conclusion, integrating nanomedicine with cell therapy, CAR-Exosomes hold significant promise as a next-generation platform aiming for high efficacy, safety, and broad clinical applicability in cancer immunotherapy.

## 1 Introduction

Engineered Chimeric Antigen Receptor (CAR) cell therapy represents a significant milestone in cancer immunotherapy. Since the approval of the first CAR-T cell product in 2017, this therapy has achieved remarkable success, demonstrating complete remission rates exceeding 80% in relapsed/refractory B-cell malignancies (1, 2). However, its application in solid tumors continues to face fundamental challenges. These include severe on-target off-tumor toxicities such as cytokine release syndrome (CRS) and neurotoxicity (3, 4), as well as physical barriers and immunosuppressive characteristics within the tumor microenvironment (TME) that impede sufficient CAR-T cell infiltration and activation (5). To address these limitations, researchers have developed CAR-NK cells (6, 7)and CAR-macrophage (CAR-M) cell therapies (8, 9). These alternative approaches offer advantages such as “off-the-shelf” potential and improved infiltration into solid tumors (10). Nevertheless, they are still limited by key issues, including short *in vivo* persistence, restricted capacity for ex vivo expansion, complex manufacturing processes, and continued suppression by the TME, which collectively diminish their anti-tumor efficacy (10).

Against this backdrop, exosomes, the natural lipid bilayer vesicles with diameters of 30–150 nm, have emerged as key to breaking this impasse owing to their unique biological properties (11). As crucial mediators of intercellular communication, exosomes are capable of carrying proteins, nucleic acids, and lipids, and can efficiently traverse physiological barriers, including the blood-brain barrier (BBB) (12, 13). Exosomes derived from CAR-engineered immune cells including CAR-T cells (14–16), CAR-NK cells (17) and CAR-M (18), which inherit the targeting specificity, low immunogenicity, and multi-drug loading capacity of their parent cells, have become an emerging hot topic in cancer immunotherapy. This review comprehensively examines the core challenges for CAR-T, CAR-NK, and CAR-M therapies and emphasizes recent advances in CAR-Exosomes, including their molecular characteristics, targeted recognition mechanisms, tumor-killing pathways, biosafety, engineering strategies, and challenges and strategies in the clinical translation.

## 2 The mechanism of action, advantages and limitations in CAR-immune cells therapies

### 2.1 The mechanism of action, advantages and limitations in CAR-T cell therapies

#### 2.1.1 The mechanism of action in CAR-T cell therapies

CAR-T cells specifically recognize and bind to tumor-associated surface antigens through their CARs, leading to full activation and extensive proliferation. Once activated, CAR-T cells efficiently kill tumor cells via multiple mechanisms (**Table 1**): release perforin and granzyme B to induce tumor cell apoptosis (19); express Fas ligand (FasL) to engage death receptors on tumor cells, triggering apoptotic signaling pathways (19); and secrete proinflammatory cytokines such as IFN-γ and TNF-α to directly suppress tumor growth or induce cell death while simultaneously recruiting and activating other immune cells to remodel the immunosuppressive tumor microenvironment (TME) (20), thereby enhancing systemic antitumor immunity.

**Table 1:** The comparisons of CAR-T, CAR-NK, and CAR-M cells.

Key Feature Indicators	CAR-T cell	CAR-NK cell	CAR-macrophage
Killing mechanism	1. Perforin/Granzyme B-induced apoptosis; 2. FasL-mediated death receptor pathway; 3. IFN-γ and TNF-α mediated indirect tumor killing coupled with tumor microenvironment remodeling.	1.Perforin/Granzyme- B induced apoptosis; 2. Death receptor (TRAIL/FasL) pathway; 3. Antibody-dependent cellular cytotoxicity; 4. Cytokine secretion (IFN-γ/TNF-α) for tumor immune microenvironment remodeling.	1. Targeted phagocytosis; 2. Release of lysosomal enzymes & ROS to induce cell apoptosis; 3. Antigen presentation: Adaptive immune activation &immunological memory formation; 4. Secretion of cytokine Chemokine (e.g., IL-12, CCL5) for immune cell recruitment and tumor microenvironment remodeling.
Target recognition	CAR Recognition	Recognition through both CAR and intrinsic NK receptors (e.g., NKG2D)	Target recognition through both CAR and macrophage-expressed immune recognition receptors.
Persistence	Lasting for a long time (several months to several years)	Short-to-medium (2–4 weeks, but some patients have detected it for up to 15 months)	Short (less than 28 days)
Clinical status	An approved treatment for certain hematologic malignancies; currently in Phase I/II clinical trials for solid tumors.	Not yet approved, but Phase I/II clinical trials (hematologic/solid tumors) show encouraging safety/efficacy data.	Phase I trials showed preliminary safety, tolerability and manufacturing feasibility.

#### 2.1.2 Advantages in CAR-T cell therapies

CAR-T cell therapies represent a revolutionary breakthrough in tumor immunotherapy. Its core advantage lies in its ability to precisely target tumor-specific antigens (such as CD19 and BCMA), demonstrating potent and durable cytotoxicity against certain hematologic malignancies that are resistant to or recurrent after traditional treatments, including B-cell leukemia, lymphoma, and multiple myeloma (21–23). Additionally, as a “living drug,” CAR-T cells can expand and persist within the body (24), especially by forming memory cells (25), providing long-term immune surveillance and enabling continuous anti-tumor activity.

#### 2.1.3 Limitations in CAR-T cell therapies

**Figure 1:**
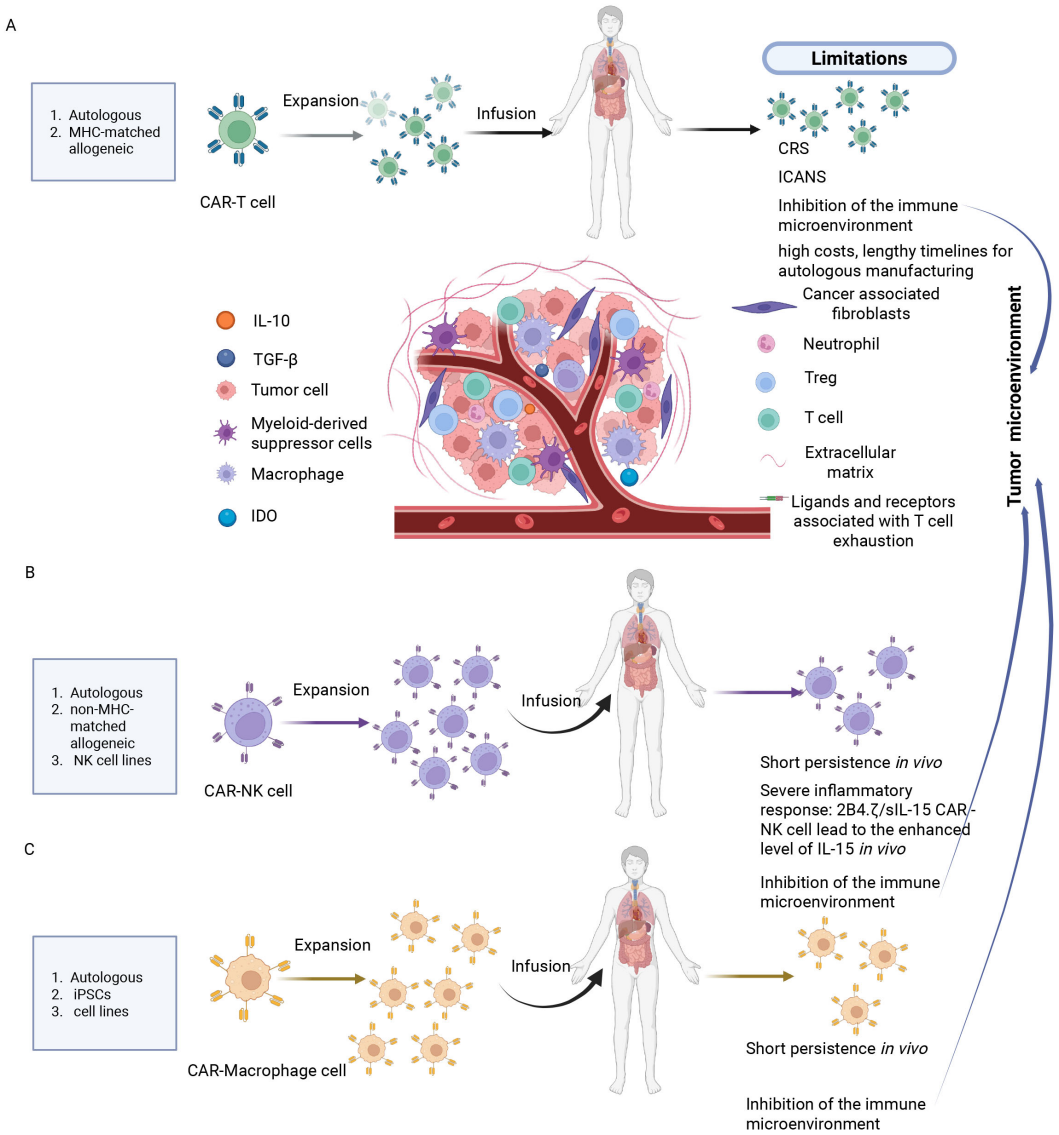
Current challenges of CAR-T, NK and macrophage therapies in solid tumor treatment. **(A)** CAR-T cell therapy: Cytokine release syndrome, immune effector cell-associated neurotoxicity syndrome, suppression of effector function by the immunosuppressive tumor microenvironment and target-dependent fratricide among CAR-T cells. **(B)** CAR-NK cell therapy: short persistence of adoptively transferred cells *in vivo*, severe immune responses such as elevated IL-15 levels induced by 2B4.7/slL-15 CAR-NK constructs, and inhibition of NK cell activity by the tumor microenvironment. **(C)** CAR-Macrophage therapy: limited persistence *in vivo*, and suppression of CAR-M cell activity by the tumor microenvironment. Created in https://BioRender.com.

However, CAR-T cell therapy also has significant limitations (**Figure 1A**). Firstly, safety concerns, including adverse reactions such as CRS, immune effector cell-associated neurotoxicity syndrome (ICANS), immune effector cell-associated hemophagocytic lymphohistiocytosis-like syndrome, nephrotoxicity, along with potential on-target/off-target toxicities (26–28); secondly, limitations in efficacy against solid tumors, primarily constrained by factors like antigen escape, limited T cell adaptability, the complex tumor microenvironment (TME), low infiltration and homing efficiency (29, 30); accessibility challenges, encompassing high costs and lengthy timelines for autologous manufacturing (31, 32).

### 2.2 The mechanism of action, advantages and limitations in CAR-NK cell therapies

#### 2.2.1 The mechanism of action in CAR-NK cell therapies

The mechanism of action of CAR-NK cell therapy centers on the fusion of the targeting capabilities of CARs with the inherent immune-killing properties of NK cells (**Table 1**). CAR-NK cells specifically recognize and bind to target antigens on the surface of tumor cells via the CAR structure they express, enabling precise targeting. Simultaneously, the activating receptors inherent to NK cells (e.g., NKG2D) can independently recognize stress-related molecules on the tumor cell surface, forming a dual-targeting recognition system and reducing the risk of antigen escape (33). Activated CAR-NK cells kill tumors through multiple effector mechanisms: releasing perforin and granzymes to directly induce tumor cell apoptosis; expressing FasL and TRAIL to trigger apoptosis of target cells via death receptor pathways; and engaging in antibody-dependent cellular cytotoxicity (ADCC) to kill tumor cells (34, 35). Moreover, upon activation, CAR-NK cells secrete cytokines such as IFN-γ and TNF-α, inhibiting tumor growth and reshaping the immunosuppressive tumor microenvironment (36).

#### 2.2.2 Advantages in CAR-NK cell therapies

CAR-NK cell therapy offers significant advantages, combining the targeting capabilities of CARs with the innate immunity of NK cells to provide dual recognition (CAR and receptors like NKG2D), potentially reducing the risk of antigen escape. This therapy utilizes a triple-killing mechanism (perforin/granzyme B, death ligand pathway, and ADCC) to achieve highly effective anti-tumor effects (33–35). Furthermore, CAR-NK cells demonstrate a superior safety profile, with a significantly lower incidence of severe cytokine release syndrome (CRS) and neurotoxicity compared to CAR-T cell therapy (37). The risk of graft-versus-host disease (GvHD) following allogeneic infusions is also notably low (38, 39). Importantly, CAR-NK cell production does not strictly rely on autologous cells. This foundation enables the development of “off-the-shelf” therapies using allogeneic sources such as the NK92 cell line (40), cord blood NK cells (41), and induced pluripotent stem cell-derived NK cells (42).

#### 2.2.3 Limitations in CAR-NK cell therapies

However, CAR-NK cell therapies still have some limitations (**Figure 1B**). Firstly, NK cells have a short lifespan *in vivo*, with a half-life of less than 10 days (43). Secondly, the genetically modified CAR-NK cells expressing IL-15/21 may cause systemic toxic reactions due to the secretion of IL-15 (6). Thirdly, the treatment of solid tumors is hindered by the tumor microenvironment barriers, such as immunosuppressive factors like TGF-β and physical stromal barriers, which result in low infiltration efficiency (44).

### 2.3 The mechanism of action, advantages and limitations in CAR-M cell therapies

#### 2.3.1 The mechanism of action in CAR-M cell therapies

CAR-M therapies utilize CARs and innate immune recognition receptors for precise recognition of tumor antigens, triggering macrophage activation and initiating multiple anti-tumor mechanisms (**Table 1**). Firstly, activated CAR-macrophages exhibit potent phagocytic activity, selectively engulfing and eliminating tumor cells (8). Secondly, CAR-M release metalloproteinases to degrade the physical barriers of solid tumors, enhancing efficacy; they also secrete cytotoxic substances such as lysosomal enzymes and reactive oxygen species, directly killing tumor cells (45). Moreover, as professional antigen-presenting cells, CAR-M cross-present the engulfed tumor antigens to CD8χ/CD4χT cells, thereby activating adaptive immune responses and establishing immunological memory (46). Finally, CAR-M secrete chemokines, to recruit and activate other immune cells, collectively constructing a more robust anti-tumor immune microenvironment, significantly amplifying the overall therapeutic effect (47, 48).

#### 2.3.2 The advantages in CAR-M cell therapies

CAR-M demonstrates strong anti-tumor potential, with key advantages including the ability to penetrate and infiltrate the tumor microenvironment. Additionally, CAR-M cells secrete various cytokines and chemokines to induce immune activation within the tumor microenvironment, thereby enhancing the anti-tumor response (47, 48). Moreover, macrophages can be derived from a wide range of sources, including human peripheral blood monocytes, iPSCs (49), and THP-1cells (50), which facilitates the development of universal CAR-M cell therapies.

#### 2.3.3 The limitations in CAR-M cell therapies

Despite the significant advantages of CAR-M cell therapy, it still faces considerable challenges (**Table 1**). Firstly, the short *in vivo* lifespan of macrophages, approximately 7 days, limits its durable anti-tumor effects (51). Of greater concern is the potential issue arising from the plasticity of macrophage phenotypes. Macrophages within the tumor microenvironment tend to differentiate into M2 phenotypes, which promote cancer growth and metastasis; this can potentially diminish the therapeutic efficacy of CAR-M cells (51) (**Figure 1C**).

## 3 The biological characteristics of CAR-Exosomes

### 3.1 An overview of CAR-exosomes

To address numerous challenges currently faced by CAR-immune cell therapy in clinical applications, CAR-Exosomes have garnered increasing attention in recent years. Exosomes are a class of extracellular vesicles, 30 to 150 nanometers in diameter, possessing unique biological characteristics (52). They originate from intraluminal vesicles (ILVs) within multivesicular bodies (MVBs). These ILVs are subsequently released as exosomes when MVBs fuse with the cell membrane via exocytosis (13). The lipid bilayer membrane of exosomes, composed of sphingomyelin, cholesterol, and lipid raft-associated proteins, confers high stability and biocompatibility (53). Exosomes carry a rich array of bioactive molecules, including proteins such as transporters and signal transduction molecules, nucleic acid such as mRNA (54) and miRNA (55), making them important intercellular communication vehicles. they are involved in regulating various physiological and pathological processes, such as immune response (56), tissue repair (57), and tumor progression (58). Their endogenous structure, naturally carried molecular components, and low immunogenicity not only provide potential therapeutic effects but also position exosomes as promising diagnostic markers and drug delivery vehicles due to their excellent biocompatibility and stability (59, 60).

Building upon the established foundation of CAR-engineered immunotherapies such as CAR-T, CAR-NK, and CAR-M cells, researchers have pioneered CAR-Exosomes, an emerging therapeutic platform (61). Functioning as a “miniature” version of their parental immune cells, CAR-Exosomes inherit the biological characteristics and material transport capabilities of their cell of origin. Critically, the functional CAR structures expressed on their surface provide the ability to precisely recognize tumor-associated antigens, thereby enabling targeted intercellular communication and drug delivery (14, 18, 62). By leveraging the natural properties of exosomes, CAR-Exosomes exhibit excellent stability, tissue migration, and penetration capabilities, allowing them to reach tumor sites more effectively (63). Furthermore, as immune cell-derived vectors, CAR-Exosomes can also modulate the immune microenvironment and enhance immune responses in the anti-tumor process (18). Compared to traditional cell therapies, exosome-based therapies generally exhibit lower immunogenicity and a reduced risk of side effects (14), thereby improving safety. Furthermore, studies indicate that CAR-Exosomes can serve as a multifunctional platform for efficient drug loading, addressing diverse therapeutic needs and offering more adaptable and precise strategies for tumor treatment (14, 15). A comparative analysis of CAR-immune cells and CAR-Exosomes is presented in **Table 2**.

**Table 2:** Comparison of CAR-immune cells and CAR-Exosomes.

Characteristics	CAR-immune cell	CAR-immune cell-derived-exosomes
Nature	Genetically engineered immune cell	Nano-sized lipid bilayer-enclosed biomolecules
Source	Patient or donor immune cells	Isolated/purified from culture supernatant of CAR-immune cells
Key components	Cell expressing Chimeric Antigen Receptor (CAR) on its membrane	CAR protein, proteins, nucleic acids (RNA, DNA), lipids, and other bioactive molecules
Size	Large (T/NK cells: 10-15 μm; macrophages ~20 μm)	Very small (30–150 nm)
Penetration of vasculature & solid tumor barriers	Difficult	Easy
Expansion & persistence	Can activate and expand *in vivo*	Does not proliferate; relatively short *in vivo*
Manufacturing	Requires cell isolation, ex vivo genetic engineering, expansion, Quality inspection, and reinfusion (autologous)	Purified from CAR-cell culture supernatant; can be lyophilized; easier to standardize and scale up. Greater potential for allogeneic “off-the-shelf” use.
Cross the blood barrier	Difficult	Easy
Toxicity risk (CRS/ICANS)	High	Low
Sensitivity/responsiveness to PDL1	Sensitive	Less responsive
Immunogenicity	High	Low
Therapeutic potential	Proven efficacy in hematological malignancies; challenges in solid tumors	Shows unique potential in solid tumors (better penetration, higher safety); acts as immunomodulator, “cell-free” therapy, drug delivery vehicle.
Development stage	Multiple approved products (hematological malignancies); relatively mature clinically	Primarily preclinical research; few in early clinical trials; emerging frontier field

### 3.2 The isolation methods of CAR-exosomes

**Figure 2:**
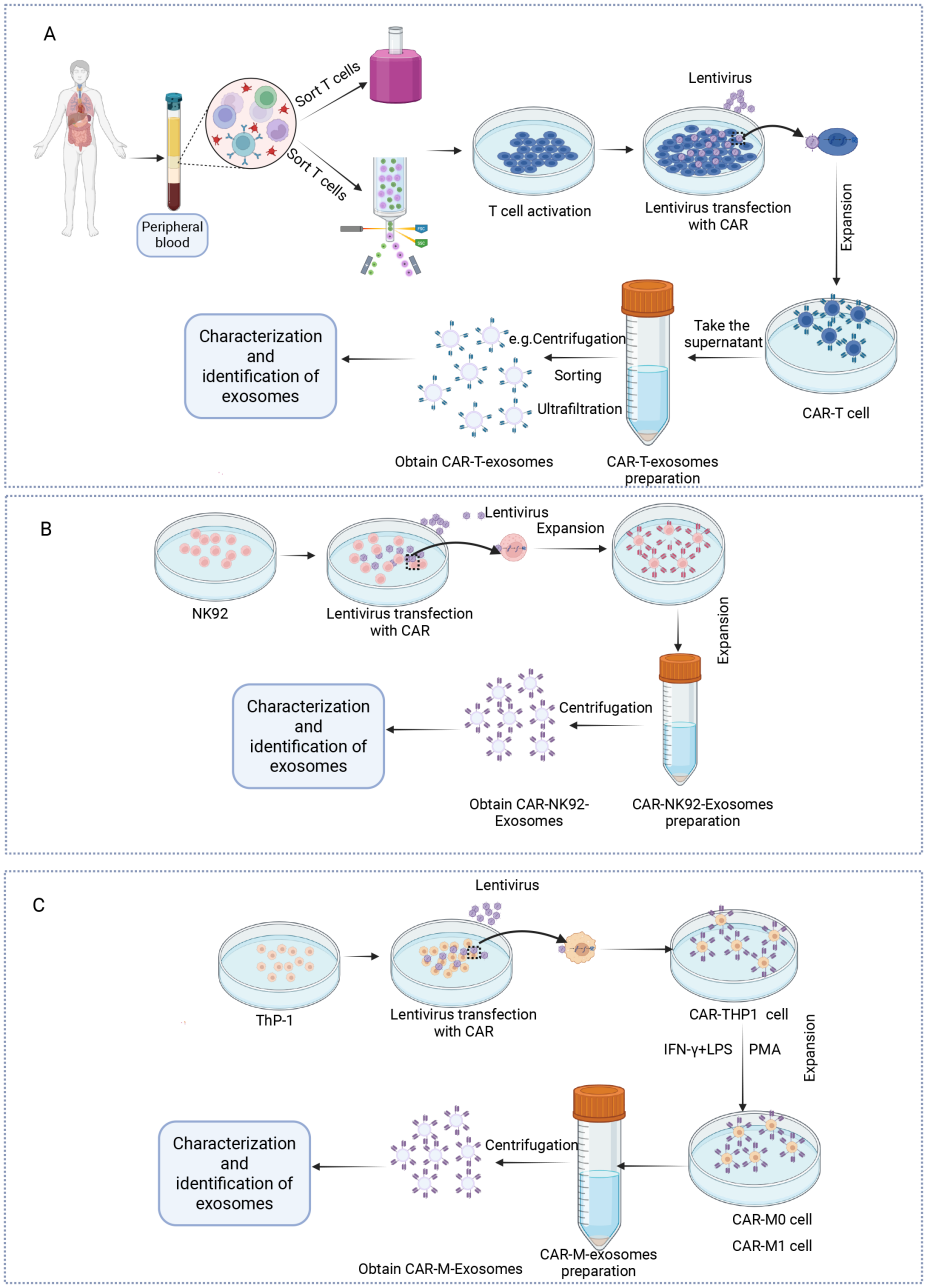
Protocol for the preparation of exosomes from CAR-T, NK and macrophage cells. **(A)** T cells are isolated from the peripheral blood mononuclear cells of healthy donors. Following activation with anti-CD3/CD28 antibodies and interleukin-2 (IL-2), the T cells are transduced with lentiviral or retroviral vectors encoding the CAR construct. The successfully transduced CAR-T cells are then expanded *in vitro* under controlled culture conditions. Exosomes are then obtained via centrifugation or other methods and subsequently characterized. **(B)** The virus carrying the CAR is transfected into NK92 cells, which are then expanded *in vitro*. Exosomes are obtained through centrifugation, followed by exosome characterization. **(C)** The virus carrying the CAR is transfected into THP-1 cells, which are then polarized into CAR-M0 or CAR-M1 cells by adding PMA or IFN-γ/LPS. Exosomes are then isolated via centrifugation and subsequently characterized. Created in https://BioRender.com.

CAR-Exosomes are derived from CAR-engineered immune cells through a standardized production process. Initially, T cells, NK cells, and THP-1 cells are genetically modified via lentiviral transduction to express chimeric antigen receptors, generating CAR-T, CAR-NK, and CAR-THP1 cells respectively. Following expansion, exosomes are isolated by ultracentrifugation (64), ultrafiltration (17), density gradient centrifugation (65), size exclusion chromatography (66), immunoaffinity capture (67), polymer precipitation (68), microfluidic techniques (69), and sorting (70), each have their respective advantages and disadvantages (**Figure 2**). We have summarized these in detail, as shown in **Table 3**. Of note is the significant potential demonstrated by emerging methods based on cell sorting technology (e.g., flow cytometry) (70, 71). Their outstanding characteristics include ultra-high specificity, high purity, low sample volume requirements, multi-parameter analysis capabilities, effective avoidance of non-target contaminants, and precise quantification accuracy. These advantages make them especially well-suited for obtaining highly purified exosomes and conducting detailed phenotypic analyses. Specifically, such cell sorting techniques also provide an effective approach for characterizing exosomes derived from CAR-T cells.

**Table 3:** The isolation methods of exosomes.

The isolation methods	Advantages	Limitations
Ultracentrifugation	Suitable for large batches; Technologically mature.	Time-consuming; Complex operation; Low yield; Potential exosomes damage.
Ultrafiltration	Quick and easy to operate; No special equipment required.	Low yield; Membrane adsorption losses; Loss of small exosomes; Membrane clogging with large volumes; Low purity.
Density gradient centrifugation	High purity; Capable of separating different vesicle subgroups; Retaining biological activity.	Cumbersome and time-intensive; Relatively low recovery rate; Requires expensive equipment and expertise.
Size exclusion chromatography	High purity; Mild temperature; High scalability; Good repeatability.	Limited resolution; Samples may be diluted; Small processing capacity; Time-consuming.
Immunoaffinity capture	Ultra-high specificity and purity; mild; suitable for low-abundance samples.	Extremely high cost; Low yield; Elution process may damage exosomes.
Polymer precipitation method	Easy to operate; Fast; Low cost; High yield; No special equipment required.	Low purity (contamination present); Polymer interferes with downstream analysis; Exosome aggregates difficult to resuspend.
Microfluidics-based techniques	Minimal sample volume required; Fast; High integration and automation potential; High purity/high recovery potential; Low shear force.	High technical complexity; Low throughput; Difficult standardization; Potentially high equipment costs.
Sorting	Ultra-high specificity; High purity; Low sample volume required; Multi-parameter analysis possible; Avoidance of non-target contaminants; Quantitative potential.	Strict parameter optimization required; trained personnel to operate required.

### 3.3 Molecular characteristics of CAR-exosomes

#### 3.3.1 The characteristics of CAR-T-exosomes

**Figure 3:**
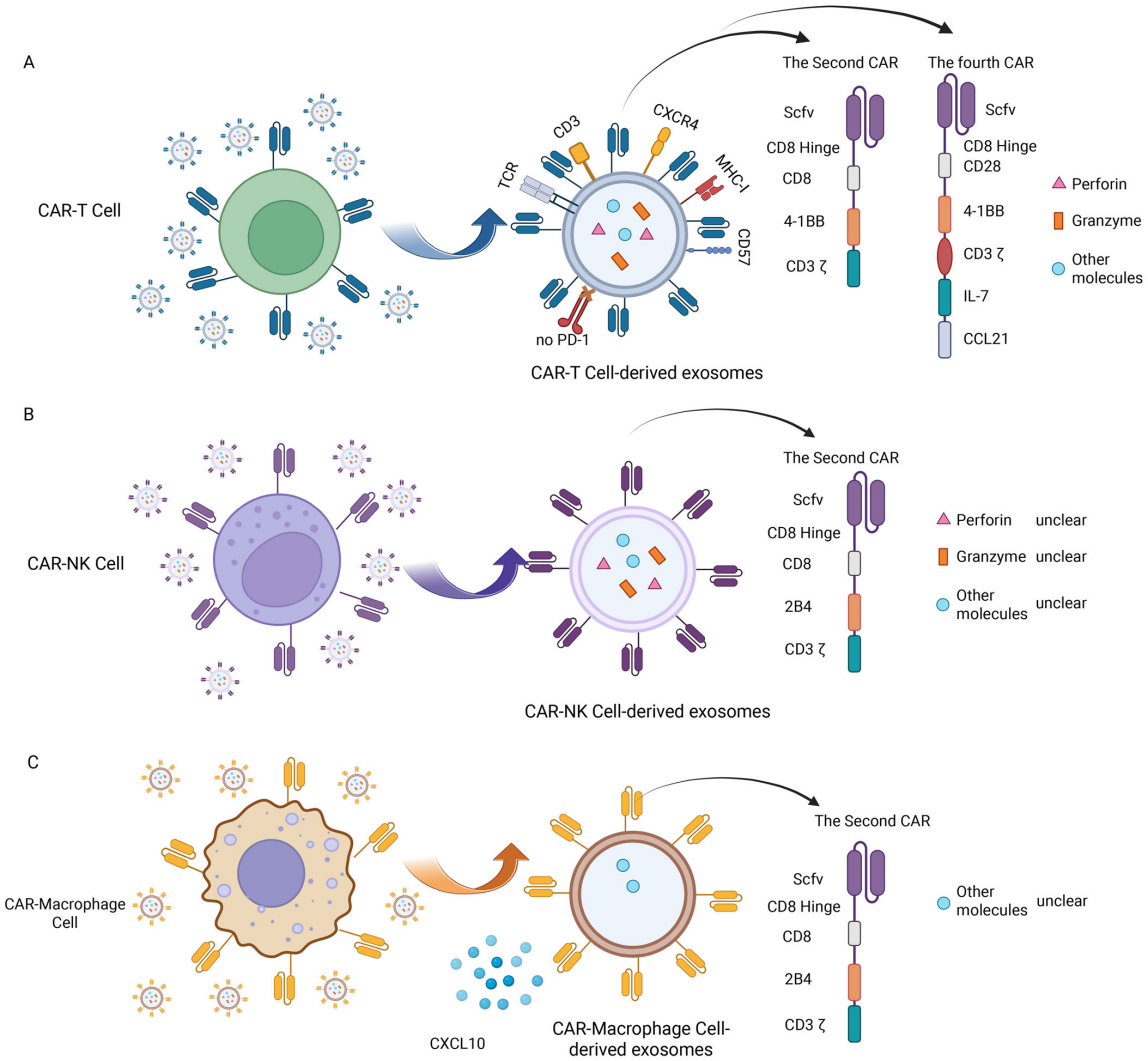
Molecular characteristics of CAR-Exosomes. **(A)** Molecular characteristics of CAR-T-Exosomes; **(B)** Molecular characteristics of CAR-NK-Exosomes; **(C)** Molecular characteristics of CAR-M-exosomes. Created in https://BioRender.com.

Comprehensive structural and molecular characterization is crucial to fully realize the therapeutic potential of CAR-Exosomes in tumor therapy. Given the frequent multi-target nature of solid tumors, CAR-T cells targeting different targets have emerged (72–75), leading to the development of CAR-Exosomes with corresponding targeting specificities. Studies on these exosomes have demonstrated that they typically carry the CAR structure from their parental CAR-T cells, alongside typical T cell markers such as CD3 and TCR (14, 16) (**Figure 3A**). To date, there have been five generations of CAR structures developed for CAR-T cell therapy. Nevertheless, for CAR-Exosomes, most published studies currently utilize second-generation CAR designs, which include an scFv^+^CD8α hinge and transmembrane domain, along with intracellular domains comprising human 4-1BB and CD3ζ (14–17). In addition, one study reported the use of a fourth-generation CAR structure, consisting of an scFv/CD8α hinge and transmembrane domain, a 2B4 co-stimulatory domain, and an intracellular CD3ζ signaling domain (76) (**Figure 3A**). For exosomes characterization, they exhibit classic exosomes characteristics and contain key effector molecules such as perforin and granzyme B (14, 63). Specifically, for example, in targeted studies against tumors with high expression of EGFR and HER2, CAR-T cells constructed using cetuximab scFv or trastuzumab scFv were both able to generate exosomes expressing CAR. Notably, CAR expression levels were higher in exosomes produced by CAR-T cells stimulated with irradiated antigen-expressing cells compared to those stimulated with CD3/CD28 antibodies. Structural analysis revealed that the CAR molecules on these exosomes exhibited the same topological structure as cell membrane CARs, with their extracellular domains exposed. Furthermore, these exosomes contained MHC class I proteins, abundant CD3, CXCR4, and CD57, but did not express CD45RA and PD-1. Additionally, perforin and granzyme B were detected (14). Similarly, exosomes derived from CAR-T cells targeting mesothelin (MSLN) (15, 16, 63) or CLDN18.2 (76) also exhibited typical exosomes characteristics, expressing anti-MSLN-CAR or anti-CLDN18.2-CAR respectively, as well as CD3 (**Table 4**).

**Table 4:** Comparisons of biological characteristics between CAR-T, CAR-NK, and CAR-M cell-derived exosomes.

The biological characteristics	CAR-T-exosomes	CAR-NK-exosomes	CAR-M-exosomes
The source of cells	human or mouse healthy donor	NK-92	THP-1
Membrane structural composition			
Membrane structural	Phospholipid bilayer structure	Phospholipid bilayer structure	Phospholipid bilayer structure
Identified exosome markers	CD63, CD9, TSG101	CD9, CD81, CD63	CD63
CAR	The second CAR-structure: scFv, CD8α hinge region and transmembrane domain, 4-1BB and CD3ζ; The fourth CAR-structure: scFv, CD8 hinge region and transmembrane domain, 4-1BB, CD3ζ, IL-7, CCL21	The second CAR-structure: scFv, CD8α hinge region and transmembrane domain, 2B4 and CD3ζ	The second CAR-structure: scFv, CD8α hinge region and transmembrane domain, 4-1BB and CD3ζ
scFV (different targets)	MSLN/PD-L1, MSLN, CLDN18.2	HER2	CD19
Other markers	High-expression of MHC I, CD3, CXCR4, CD57, TCR; low expression of CD17 receptor and CD28; undetectable expression of CD45 RA and PD-1	CD56, CD16 (FcγRIIIc), NKp46, NKG2D, KIRs?	CD14, CD11b, FcγR?
Cargo content			
Effector molecules	Perforin, granzyme	Perforin, granzyme?	lysosomal enzyme?
Cytokines or chemokines	INF-γ, TNF-α, CXCL19?	INF-γ, TNF-α, CCL1/2/20?	CXCL10 (reported), TNF-α, IL-10, IL-1β?
Nucleic acid or metabolite	no reported	no reported	no reported

#### 3.3.2 The characteristics of CAR-NK-exosomes

Beyond CAR-T-Exosomes, CAR-NK cell-derived exosomes (CAR-NK-Exosomes) are emerging as a promising therapeutic alternative, combining the advantages of CAR-NK cells while potentially overcoming their inherent limitations (**Table 4**). Theoretically, CAR-NK-Exosomes have stronger tumor tissue penetration and may be able to penetrate deeper into tumors, as well as potentially exhibit lower systemic toxicity. However, current research remains relatively limited, and their molecular characteristics are poorly understood. In one study, researchers utilized the NK92 cell line, and after transfecting it with a second CAR structure targeting HER2 (**Figure 3B**), successfully prepared CAR-NK-Exosomes expressing anti-HER2 scFv, while maintaining their typical exosomes phenotype (17). However, other components of exosomes have not yet been identified, such as perforin, granzyme B, surface receptors and some specific molecules (**Figure 3B**). Therefore, future studies are warranted to elucidate the characteristics of CAR-NK-Exosomes.

#### 3.3.3 The characteristics of CAR-M-exosomes

Following CAR-T and CAR-NK cells, CAR-M-derived exosomes (CAR-M-Exosomes) have recently gained attention as a novel extracellular therapeutic tool, based on the unique advantages of CAR-M in tumor recognition, microenvironment penetration, and immune remodeling (77, 78). One preliminary study indicates that CAR-M targeting CD19 secrete exosomes carrying CAR proteins from the parent cells and possessing typical exosomes phenotypes (**Table 4**). In this study, the CAR structure within CAR-M is a second-generation CAR. Proteomic analysis further demonstrated that the expression of chemokine CXCL10 was significantly upregulated in CAR-M-exosomes compared to control exosomes lacking CAR transfection, a characteristic potentially inherited from the highly effective immunomodulatory capabilities of CAR-M cells (18) (**Figure 3C**). However, the surface markers (such as macrophage cell-associated receptors) have not been identified, and more detailed molecular characterization of CAR-M-Exosomes requires further investigation.

## 4 Antitumor effects and anti-third party activity of CAR-Exosomes

### 4.1 Antitumor effects and mechanisms of CAR-exosomes

#### 4.1.1 Antitumor effects and mechanisms of CAR-T-exosomes

**Figure 4:**
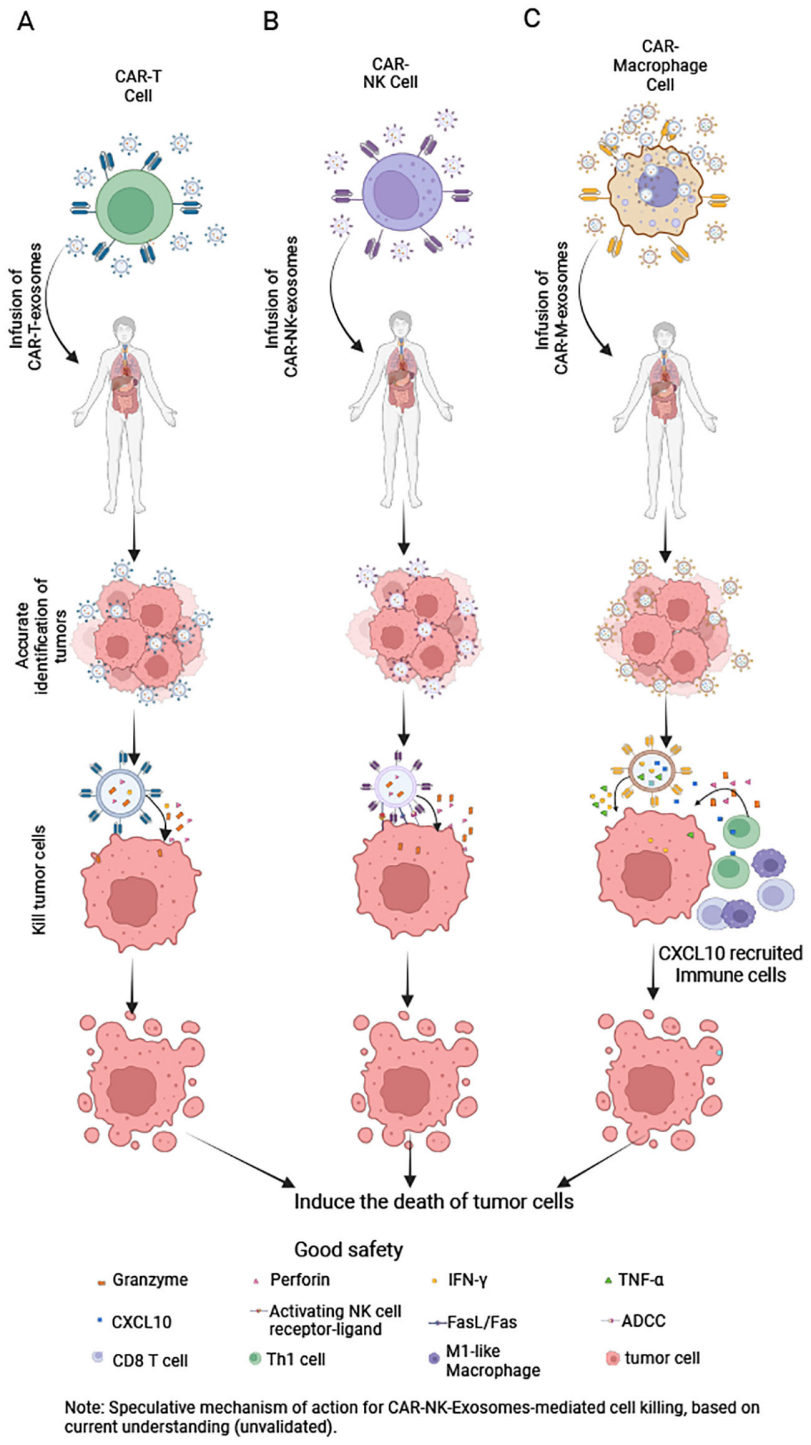
Mechanisms of CAR-Exosomes-mediated tumor cell killing. **(A)** CAR-T-Exosomes: After CAR recognizes tumor cells, they kill them via perforin and granzyme **(B)**. **(B)** CAR-NK-Exosomes: Upon CAR-mediated tumor recognition, they may kill tumor cells through: NK cell-activating receptors, antibody-dependent cellular cytotoxicity, FasL/Fas pathways and release of perforin and granzyme **(B)** (Note: This mechanism remains hypothetical, as no studies have yet confirmed it.). **(C)** CAR-M-Exosomes: Following CAR-dependent tumor targeting, they may exert antitumor effects by releasing IFN-γ and TNF-α to directly kill tumor cells and secreting CXCL10 to recruit immune cells into the tumor microenvironment for enhanced immune-mediated killing. Created in https://BioRender.com.

CAR-T-Exosomes express functional CARs with specific scFv, enabling them to selectively recognize tumor cells expressing specific antigens. For instance, CAR-T-Exosomes targeting EGFR/HER2 selectively bind to tumor cells that highly express these antigens, leaving cells with low or no expression unaffected (14). Furthermore, CAR-T-Exosomes targeting MSLN exhibit strong affinity for MSLN-positive triple-negative breast cancer (63) and non-small cell lung cancer cells (15). In a pancreatic ductal adenocarcinoma model, CLDN18.2-targeted CAR-T-Exosomes specifically eliminate tumor cells without harming CLDN18.2-negative cancer-associated fibroblasts (76). For CAR-T-Exosomes, upon CAR-mediated recognition of target antigens, release perforin and granzymes (**Table 5**), triggering apoptosis in target cells and demonstrating significant cytotoxicity both *in vitro* and *in vivo* (14, 76) (**Figure 4A**). Optimization of these exosomes through functional studies revealed that those generated from CAR-T cells stimulated with irradiated and homologous tumor cells (e.g., PC-9) exhibit enhanced cytotoxic efficacy (16). Importantly, while PD-L1 suppresses CAR-T cell function via interactions with PD-1 (14, 79), the anti-tumor activity of CAR-Exosomes appears unaffected, as demonstrated in xenograft model (14). Furthermore, the finding that CAR-Exosomes derived from HEK293T cells (cells which lack cytotoxic granules) transfected with anti-CD19 CAR are able to selectively kill CD19χ target cells, while remaining ineffective against CD19-negative cells (62). This observation strongly suggests the involvement of non-canonical cytotoxic mechanisms in CAR-Exosomes, highlighting the need for further investigation into unidentified effector molecules and their underlying pathways. Further research is needed to elucidate these mechanisms in the future.

**Table 5:** Comparisons of different CAR-Exosomes.

The key characteristics	CAR-T-exosomes	CAR-NK-exosomes	CAR-M-exosomes
Recognition mechanism	CAR-dependent targeting	CAR-specific binding(verified); Engagement of natural cytotoxicity receptors (e.g., NKG2D, NKp46) (unverified)	CAR-mediated specificity (verified); Pattern recognition receptors (e.g., TLR4) (unverified)
Killing mechanism	Perforin/granzyme B-induced apoptosis	Perforin/granzyme B (unverified) FasL/TRAIL-mediated apoptosis (unverified) Antibody-dependent cellular cytotoxicity (unverified).	CXCL10-mediated immune cell recruitment; Metalloproteinases (e.g., MMP-9)-dependent matrix degradation (unverified); Lysosomal enzyme delivery (unverified).
The ability to penetrate tumor tissues (relative to CAR-immune cells)	Easy	Easy	might be easier compared to CAR-T/NK-Exosomes, based on the inherent migration and penetration capabilities of macrophages
Production difficulty	High	Low	Medium

#### 4.1.2 Antitumor effects and mechanisms of CAR-NK-exosomes

Furthermore, CAR-NK-Exosomes not only recognize tumor cells via their CAR but may also do so through NK cell activating receptors (80), a mechanism that warrants further investigation (**Table 5**). One study indicates that HER2-CAR-based NK cell-derived exosomes can recognize HER2-positive brain metastatic tumors (17). Upon recognizing tumor cells, CAR-NK-Exosomes may induce target cell death via several mechanisms: inducing apoptosis via perforin and granzymes, activating death receptor pathways via ligands such as FasL and TRAIL, and ADCC (33, 81, 82) (**Figure 4B**). Currently, studies on CAR-NK-Exosomes are limited. One study reported that CAR-NK-Exosomes loaded with ferroptosis inducers effectively killed breast cancer cells under light irradiation. However, this effect was dependent on exogenous drug-triggered ferroptosis pathways, and the intrinsic cytotoxic mechanisms remain incompletely understood (17).

#### 4.1.3 Antitumor effects and mechanisms of CAR-M-exosomes

Similarly, exosomes derived from CD19-CAR-M cells can rapidly and specifically adhere to Raji cell membranes expressing CD19 (18). Besides, the CAR-M-Exosomes may also recognize tumor cells through their own receptors [such as innate pattern recognition receptors (83)], and this phenomenon requires further investigation. Building upon the well-established cytotoxic mechanisms of CAR-M cells (51, 84, 85), we hypothesize that CAR-M-Exosomes may mediate anti-tumor effects through the release of lysosomal enzymes, and other effector molecules (**Table 5**). Currently, research on CAR-M-Exosomes is limited. One study demonstrated that CD19-CAR-M-Exosomes loaded with SN38 exhibit significant cytotoxic effects of lymphoma cells both *in vitro* and *in vivo*. However, the direct cytotoxic mechanism against tumor cells remains unclear. Notably, the anti-tumor activity observed in SN38-free CD19-CAR-M-Exosomes was limited, potentially due to the concentration of endogenous effector molecules within the exosomes being below the threshold required for lymphoma cell killing (18). Further studies are needed to elucidate these mechanisms in greater detail.

### 4.2 Anti-third party activity of CAR-exosomes

The ‘anti-third-party activity’ of exosomes is complex and multifaceted, encompassing both beneficial anti-tumor effects and significant safety considerations. The beneficial anti-tumor effects are as follows. First, recruitment and activation of immune cells: for example, a current study has shown that CAR-M-Exosomes not only target tumor cells but also, being rich in the chemokine CXCL10, can effectively recruit T cells and M1-type macrophages. Specifically, CAR-M-Exosomes significantly enhance T lymphocyte activation and migration, promoting their differentiation into CD8^+^T cells (18). Furthermore, CAR-M-Exosomes increase the proportion of M1 macrophages (18), which are known to have anti-tumor properties (86) (**Figure 4C**). However, further research is needed to fully understand the impact of CAR-Exosomes on other immune cells. Second, potential effects on other stromal cells: we hypothesize that if other cells in the tumor microenvironment, such as cancer-associated fibroblast or endothelial cells, express the same antigen as the target tumor cells on their surface, CAR-Exosomes could similarly recognize and directly exert a killing effect on them. Third, microenvironment modulation via cargo: CAR-Exosomes themselves may carry various bioactive molecules (e.g., cytokines, non-coding RNAs). We speculate that by releasing these substances in the TME, they could directly modulate various neighboring cell types and reshape the immunosuppressive microenvironment. It is noteworthy that this potential ‘anti-third-party activity’ also raises important safety considerations: beyond acting on the tumor site, whether circulating CAR-Exosomes might affect normal tissue cells (e.g., by expressing low levels of the target antigen or via off-target effects) leading to non-specific damage. This is a crucial question that needs to be clarified in future research.

## 5 CAR-exosomes exhibit low toxicity

The safety profile of CAR-Exosomes is an important aspect alongside their anti-tumor efficacy. Unlike traditional CAR-T cell therapies, which are often associated with severe adverse effects such as CRS and ICANS (87), CAR-Exosomes have demonstrated a significant safety advantage. Multiple preclinical studies provide compelling evidence as shown in **Table 6**. For example, a 13-week repeated-dose toxicity study, administering 25 μg, 50 μg, 100 μg, and 150 μg weekly/dose, showed that mice even at the highest dose of 150 μg did not exhibit signs of toxicity. Further research indicated that even when using T1E-CAR-Exosomes targeting ErbB dimer, administered via intraperitoneal injections at doses up to 125 mg/kg, no behavioral or body weight abnormalities were observed, and there was no detection of human pro-inflammatory cytokines such as IFN-γ and IL-2 (14). Regarding overall toxicity control, MSLN-targeted CAR-Exosomes administered intravenously at doses of 100, 300, and 500 μg on days 3, 6, 9, 12, and 15 in mice showed no signs of toxicity even at the highest dose of 500 μg during breast cancer treatment (63). Similarly, in pancreatic cancer models treated with CLDN18.2-targeted CAR-Exosomes, serum levels of IL-6, a key driver of CRS, did not increase (76). In a lung cancer model, inhaled MLSN-CAR-Exosomes loaded with PTX (20 mg/kg, inhaled nebulization for 14 days) had minimal impact on peripheral blood levels of IL-2, IL-6, and IFN-γ, which are critical components of cytokine storms (15). Additionally, histological examination of major organs such as the heart, liver, spleen, lungs, and kidneys revealed negligible toxic damage following treatment with CAR-NK-Exosomes loaded with ferroptosis inducers (specific dose unknown) for breast cancer (17). In a mouse model of lymphoma, treatment with CD19-CAR-M-Exosomes loaded with SN38 (2×10^11/week, for four doses) did not result in significant weight loss or other signs of toxicity (18). In summary, these preclinical studies suggest that CAR-Exosomes, while maintaining anti-tumor activity, possess favorable safety profiles, even at the highest tested doses, with no relevant toxicity observed.

**Table 6:** The dose and toxicity of CAR-Exosomes in preclinical studies.

Exosome-derived cells	Carry drugs	Targets	Dose regimen	Safety assessment	Route of administration	Cancer type
CAR-T	None	MSLN	100,300, 500 μg (Days 3/6/9/12/15)	No obvious toxicity was observed even at a high dose of 500 μg	Intravenous injection	Breast cancer
CAR-T	None	CLDN18.2	40 μg each time, administered every other day for 5 times	No significant increase in IL-6	Intravenous injection	Pancreatic cancer
CAR-T	Fuse with liposomes and loading paclitaxel	MSLN/PDL1	400 μg per three days, for three consecutive times	No obvious toxicity of the liver, spleen, heart, lungs and kidneys	Intravenous injection	Metastatic lung cancer
CAR-T	None	CTX/TTZ	25, 50,100, 150 μg per week for a total of 4 to 5 weeks	No signs of toxicity even at a high dose of 150 μg	Intravenous injection	Lung cancer, breast cancer and ovarian cancer
CAR-T	Load paclitaxel	MSLN	20mg/kg, nebulized inhalation for 14 days	No significant change in IL-2/IL-6/IFN-γ	Inhalation	Non-small cell lung cancer
CAR-NK	Load RAS-selective lethal-3	HER2	The dosage was not explicitly stated	No significant changes in liver and kidney functions. The morphological evaluations of the heart, liver, spleen, lungs and kidneys showed negligible toxicological lesions.	Intravenous injection	Breast cancer brain metastasis
CAR-M	Load SN-38	CD19	2×10¹¹/ week, for 4 times	No obvious signs of weight loss or toxicity	Intravenous injection	Lymphoma

However, the current preclinical studies have certain limitations. The dose range assessment of CAR-Exosomes is not comprehensive enough, lacking systematic dose escalation studies and maximum tolerated dose (MTD) data. Furthermore, differences in administration schedules, routes, and frequencies restrict the comprehensive assessment of the safety and efficacy of CAR-Exosomes. Although one study showed no significant toxicity in mice after repeated administration for 13 weeks (14), the long-term effects on the host immune system have not been fully explored. In addition, the metabolic data of CAR-Exosomes *in vivo* are currently unclear. In order to enable the clinical application of CAR-Exosomes, it is necessary to first address these limitations through preclinical studies. This includes conducting systematic dose-ranging explorations, such as determining the MTD of different types of CAR-Exosomes through dose escalation experiments. Long-term toxicity studies are also needed to evaluate the safety of repeated doses, including effects on the hematopoietic system, liver, kidneys, and immune system. Determining the optimal administration regimen is also crucial, and this should involve comparing the efficacy and safety of different administration routes, frequencies, and doses. In addition, conducting comprehensive pharmacokinetic/pharmacodynamic (PK/PD) studies to elucidate the *in vivo* distribution, clearance, targeting, and biological effects of CAR-Exosomes will be very important for guiding clinical dose selection. In conclusion, while the existing data are optimistic, more in-depth research is still needed to more comprehensively assess safety and lay a solid foundation for future clinical trials.

## 6 Engineering strategies to enhance the functionality of CAR-exosomes

Although CAR-Exosomes have significant therapeutic potential, further improvements are still needed to enhance the therapeutic effect. As natural nanocarriers, their mechanism of action primarily relies on singular targeting and signal transduction pathways, which may prove inadequate against complex tumor microenvironments or multidrug resistance. To achieve more precise and multifunctional therapeutic outcomes, conferring novel functionalities to CAR-Exosomes is crucial, including multi-targeting capabilities, multi-drug loading capacity, and immunomodulatory effects. These limitations necessitate the adoption of engineered modification strategies, involving systematic design and optimization, to improve CAR-Exosomes performance and broaden their application potential, thereby better addressing diverse clinical therapeutic needs.

### 6.1 Parental cell engineering

CAR-Exosomes can inherit certain characteristics from their parent cells, such as CAR-T, NK, and macrophage cells. Consequently, modifying the parental cells represents a key strategy for engineering CAR-Exosomes. This approach includes employing genetic editing technologies to generate dual-target CARs, which can further mitigate tumor antigen escape and enhance the activation and function of CAR-T cells (88). Existing studies have validated the efficacy of various dual-targeting CAR-T cells, including CD19/BCMA CAR-T (89), BCMA/GPRC5D CAR-T (90), and B7-H3/CSPG4 CAR-T cells (91), which have laid a critical foundation for developing multi-targeted CAR-Exosomes. In a study focused on lung cancer treatment, researchers developed dual-target CAR-T cells simultaneously targeting MSLN and PD-L1. Exosomes derived from these cells can precisely target MSLN-positive tumor cells. Furthermore, the anti-PD-L1-scFv within the exosomes blocks PD-L1 in the tumor microenvironment, reducing the exhaustion of infiltrating T cells (16). Furthermore, this involves optimizing the CAR structure to enhance T cell activation, proliferation, persistence, and cytotoxic capacity (51). CAR-T cells can also undergo metabolic reprogramming; for instance, overexpression of glucose transporter type 1 in CAR-T cells induces metabolic reprogramming and enhances efficacy (92). These optimized traits may also be reflected in the secreted CAR-Exosomes. Therefore, modification of parental cells enables the engineering of their derived exosomes. In summary, by modifying the parental cells, researchers can systematically tailor the biological properties of CAR-Exosomes, leading to more potent and multifunctional therapeutic applications.

### 6.2 Post-isolation engineering

#### 6.2.1 Physical modification and drug loading

The drug loading of Exosomes primarily relies on physical mechanisms such as non-covalent interactions, diffusion, and membrane permeability changes to efficiently incorporate therapeutic molecules like nucleic acids and small-molecule drugs into the interior of the exosomes (52). The most used method, co-incubation/passive diffusion, involves incubating the drug molecules with exosomes under optimized conditions of temperature, pH, and buffer solutions, with continuous stirring (93–95). During this process, drugs passively diffuse into the exosomes driven by concentration gradients or bind to the membrane via hydrophobic interactions. Subsequently, free drugs are removed through dialysis, ultrafiltration, or size exclusion chromatography (16, 93, 96, 97). For instance, in studies focused on lung cancer therapy, PTX was co-incubated with Liposome-CAR-T-Exosomes targeting MSLN/PD-L1 for 40 minutes, followed by dialysis purification, successfully generating a Liposome-CAR-T-Exosomes@PTX complex exhibiting therapeutic effects (16). Additionally, other loading methods include electroporation, extrusion and sonication methods (98, 99). Research demonstrates that electroporation can efficiently load PTX into CAR-Exosomes targeting MSLN, yielding not only favorable drug release characteristics but also targeted lung distribution and substantial tumor growth inhibition in lung cancer models (15). These physical loading methods, due to their simplicity and ability to preserve exosome integrity, offer critical technical support for the clinical application of CAR-Exosomes.

#### 6.2.2 Membrane fusion engineering

Membrane fusion technology offers a novel approach to functional optimization of CAR-Exosomes. The thin-film hydration method, which induces membrane reorganization between CAR-Exosomes and functionalized liposomes, enables the construction of hybrid vesicles with dual properties (16). For targeted therapy applications, researchers have addressed the hepatic accumulation limitation of conventional CAR-T-Exosomes by fusing them with lung-targeting liposomes via thin-film hydration, successfully developing a novel hybrid vesicle termed Lipsomes-CAR-Exosomes. This innovative structure not only retains the original tumor-targeting capability of CAR-Exosomes but also significantly enhances pulmonary accumulation efficiency by incorporating the lung-homing properties of liposomes, thereby providing a superior delivery vehicle for targeted treatment of lung cancer and other pulmonary diseases. This technology demonstrates the unique advantages of membrane fusion engineering in optimizing the tissue distribution characteristics of exosomes.

#### 6.2.3 Biomimetic conjugation strategy

The biomimetic conjugation strategy has facilitated the development of an innovative CAR-M-derived exosome-drug conjugate system that skillfully combines targeted delivery with combined chemo-immunotherapy. Employing exosomal CD63 as an anchoring point, researchers initially covalently conjugated anti-CD63 antibodies to the topoisomerase inhibitor SN38. Subsequently, through CD63–anti-CD63 binding interactions, these conjugates were co-incubated with exosomes to generate SN38-loaded CAR-M-Exosomes (18). This sophisticated system operates through a dual mechanism: the CAR component facilitates precise tumor cell targeting and internalization via receptor-mediated endocytosis, while simultaneously delivering therapeutic payloads-SN38 mediates direct antitumor activity and endogenous CXCL10 within CAR-M-Exosomes provides immunomodulatory functions. This combined approach results in significantly enhanced antitumor activity, demonstrating how biomimetic strategies can create multifunctional exosome-based therapeutics that leverage both engineered and inherent biological properties, leading to improved cancer treatment. The CD63 anchoring method provides a robust platform for developing next-generation exosome delivery systems with precisely controlled drug loading and preserved biological function.

### 6.3 Construction of multifunctional intelligent delivery systems

The advancement of CAR-Exosomes technology is being driven by multi-strategy synergistic engineering approaches. The newly developed ExoCAR/T7@Micelle intelligent delivery system successfully achieves spatiotemporally precise regulation of exosomal drugs through the integration of three core technologies: CAR targeting, T7 peptide-mediated membrane fusion, and thermosensitive micelle encapsulation, charting a course for next-generation smart delivery platforms. On the one hand, it utilizes T7 peptides to mediate efficient BBB penetration and employs CAR for precise tumor cell targeting. On the other hand, it incorporates a light-responsive amphiphilic copolymer, mPEG-TK-Ce6, to form a micellar core encapsulating the ferroptosis inducer RSL3, enabling controlled drug release upon specific light irradiation via Ce6-triggered TK bond cleavage. The experimental results demonstrate that this intelligent system exerts an anti-tumor effect by inducing ferroptosis in HER2-positive BCBM cells. It effectively addresses key limitations of conventional chemotherapy, such as poor BBB penetration, inadequate targeting, and severe side effects, offering a highly promising new strategy for precision cancer therapy with substantial clinical translation potential (17).

## 7 Clinical translation of CAR-exosomes: challenges and future directions

Although CAR-Exosomes derived from CAR-T, CAR-NK, and CAR-macrophages have demonstrated significant preclinical anti-tumor targeting activity, relatively low toxicity potential, excellent immunomodulatory capabilities, and flexible engineering potential, their progression toward clinical application still faces several key challenges.

### 7.1 Large-scale production

One of the primary challenges facing exosomes-based therapies is the low yield and efficiency of production. Exosome yields are typically less than 1 μg per milliliter of culture medium, whereas many studies require doses ranging from 10 to 500 μg of protein per mouse (100, 101). A clinical trial has indicated that each patient may require between 0.5 to 1.4 x 10^11 exosomes (102). To meet the demands of animal experiments and clinical trials for large quantities of exosomes, it is crucial to enhance the efficiency of their large-scale production. In recent years, researchers have focused on hollow fiber bioreactors and stirred-tank bioreactors to improve exosome yields (93, 103). For example, GMP-grade bioreactors, such as those from Quantum, have successfully produced large quantities of exosomes carrying Kras-G12D siRNA, demonstrating their potential to inhibit tumors in a pancreatic cancer model (104). Furthermore, another study reported a ~6-fold increase in exosomes concentration and ~3-fold increase in exosomes production rate when using MSC cells cultured on microcarriers in a vertical wheel bioreactor (similar to a stirred-tank bioreactor) with xenobiotic-free medium (102). Concurrently, techniques like differential centrifugation (105) and density gradient centrifugation (106) are commonly used for exosomes purification to increase purity; however, these methods have limited throughput and are insufficient for meeting the demands of large-scale clinical production. To address these issues, tangential flow filtration (TFF) presents itself as a promising purification technique (107). By allowing the culture supernatant to flow along the membrane surface, TFF reduces membrane fouling, and improves throughput and recovery, thereby enabling efficient separation, which is better suited for the large-scale production of exosomes.

The advancement of Artificial Intelligence (AI) is poised to accelerate the large-scale production of exosomes. In the future, machine learning models can be employed to analyze the transcriptomic, proteomic, and other omic data of high exosome-producing cell lines, predict their secretion capacity, and thus assist in cell line selection. Reinforcement learning algorithms can be utilized to dynamically adjust bioreactor parameters, including pH, dissolved oxygen, and stirring speed, enabling closed-loop optimization. Furthermore, AI can be applied for automated analysis of exosomes size and morphology, facilitating standardized characterization. Machine learning can also be employed to evaluate key indicators of different exosomes batches for quality control purposes. By combining AI for optimization across the entire production workflow, from cell preparation and media formulation to bioreactor operation and purification, it is possible to significantly reduce costs and improve production efficiency, thereby enhancing the accessibility of exosomes therapies. Therefore, integrating advanced bioreactor technology and tangential flow filtration with AI techniques holds promise for significantly increasing exosomes yield and quality, accelerating the progress towards clinical application.

### 7.2 Storage stability and activity retention

Storage stability and activity retention are critical factors impacting the performance of exosomes-based therapeutics. Therefore, investigating effective preservation techniques to protect their biological activity, facilitate transportation, and enable clinical application is of paramount importance. Currently, common preservation methods include cryopreservation, lyophilization (freeze-drying), and spray drying (108). Among these, -80°C cryopreservation is considered the optimal long-term storage solution, effectively maintaining the stability and activity of exosomes (109, 110). Research has shown that GMP-grade targeted exosomes retained significant anti-tumor activity after six months of storage at -80°C (104), while exosomes derived from milk exhibited no change in physical properties after four weeks of storage at -80°C, with the stability of loaded drugs also being maintained (111). Furthermore, incorporating cryoprotectants such as human albumin and trehalose can effectively reduce membrane damage caused by ice crystal formation, further enhancing stability (109).

In the future, researchers can leverage AI models to analyze vast storage experimental datasets and automatically optimize cryopreservation conditions, including temperature, cryoprotectant type and concentration, and buffer formulations, thereby establishing optimal storage protocols for CAR-Exosomes. Based on omics data and characterization information, machine learning models can also predict the impact of different storage conditions on exosomes stability and quality, allowing for early identification of potential risks. Additionally, customized storage and transportation strategies can be designed, tailoring them to the source, cargo, and intended application of CAR-Exosomes, thereby improving inter-center and inter-batch reproducibility and enhancing the efficiency of clinical translation.

### 7.3 Complexity in understanding mechanisms of action and composition

Despite promising preclinical results demonstrating significant anti-tumor effects of CAR-Exosomes, the precise mechanisms underlying their anti-tumor activity remain largely unelucidated. This includes how they activate the host immune system, kill tumor cells, and remodel the TME using signaling molecules. Furthermore, there is a lack of in-depth understanding of their *in vivo* behavior, including their biodistribution and interactions with target cells. Concurrently, the core bioactive components driving their activity, such as specific proteins, RNA, and lipids, have not been fully characterized. To systematically decode these complexities and promote clinical translation for targeting solid tumors, one can utilize multi-omics approaches (e.g., transcriptomics, proteomics, lipidomics), high-throughput functional assays, and animal studies to comprehensively elucidate exosomes composition, targeting properties, and the specific pathways involved in activating host immunity, killing tumor cells, and remodeling the microenvironment. For example, a study identified CXCL10 as the most enriched chemokine through proteomic identification of CAR-M-Exosomes and subsequent enrichment analysis, revealing that CAR-Exosomes exhibit significant immunomodulatory functions (18), providing important clues for further research on immune activation mechanisms. In addition, *in vivo* imaging and tracking techniques (112) such as BLI, MRI, PET, and SPECT can be used to study their *in vivo* distribution, duration, targeting, and metabolic pathways in detail.

With the advancements in AI technology, it is becoming possible to integrate the above multi-source data using AI machine learning and deep learning models to establish dynamic, multi-layered mechanism-of-action models. For instance, AI can be employed to analyze CAR-Exosomes proteomic data, as shown in a study that identified and validated serum exosomes proteomic features based on machine learning. Furthermore, AI can analyze changes in transcriptomic, proteomic, and metabolomic data following co-culture of CAR-Exosomes with tumor cells/immune cells, thereby revealing the signaling pathway regulatory mechanisms underlying CAR-Exosomes treatment of cancer. This approach can also clarify which signaling pathways the CAR-Exosomes utilize to affect immune cell activation. By inputting exosomes design parameters and synthesizing the results from previous animal experiments, it is also possible to predict their distribution in tumor tissues and other organs, and to devise exosomes administration protocols.

### 7.4 Further research on safety is required

Clinical translation of CAR-Exosomes therapies faces significant safety challenges, necessitating systematic safety validation. Currently, research primarily relies on murine models, lacking systematic assessment of toxicology, pharmacokinetics (PK), and pharmacodynamics (PD) in non-human primates (NHPs). Before clinical application, the safety issues of primary concern for CAR-Exosomes therapies include immunogenicity, long-term organ toxicity, and off-target effects. To address these, high-potential translational strategies include: assessing the effects of CAR-Exosomes on the immune system, including monitoring the distribution of lymphocyte subsets and cytokine profiles; conducting systematic toxicological, PK, and PD studies in NHPs to comprehensively evaluate the safety of the exosomes and their potential risks. While one study demonstrated that intravenous administration of 3.85×10¹² human mesenchymal stem cell-derived exosomes did not elicit overt toxic reactions (113), the safety of engineered CAR-Exosomes in patients with tumors warrants further in-depth investigation. Simultaneously, conducting dose-escalation studies to determine the safe dosage of CAR-Exosomes for tumor treatment is crucial. Furthermore, utilizing whole-body imaging techniques like PET-CT (114) to track the distribution of CAR-Exosomes in animal models will allow assessment of off-target accumulation and potential toxicities. Moreover, designing CAR activity “switch” systems with drug or signaling control can reduce the risk of non-target toxicity when necessary, thereby improving overall safety. For instance, one study showed the use of protease-regulated CARs as a “switch” for CAR-T cells (115), deactivating CAR activity upon detection of potential toxic responses, ensuring treatment safety. Additionally, CAR-T-Exosomes can be engineered in combination with biomaterials to achieve localized or targeted delivery, mitigating toxicity to other organs. For example, fusion of CAR-T-Exosomes with lung-targeting liposomes can achieve drug enrichment within the lungs, reducing systemic side effects (16).

Furthermore, AI technology is expected to empower multi-level safety defenses. At the molecular design stage, AI can utilize tools such as AlphaFold to predict scFv off-target epitopes and immunogenicity, optimizing the stability of CAR structures and enhancing their safety. For *in vivo* behavior monitoring, intelligent quantification of exosome distribution and tumor enrichment efficiency can be achieved through the fusion of multi-modal imaging (PET/MRI/optical) technologies. Concurrently, deep learning can be used to construct toxicity prediction models by integrating observed toxic reactions and corresponding laboratory abnormalities. For instance, in CAR-T research, a study successfully predicted the occurrence of ICANS in 204 patients with follicular lymphoma or diffuse large B-cell lymphoma treated with axicabtagene ciloleucel by constructing a machine learning-based logistic regression model (116). CAR-Exosomes therapies can learn from the experiences of CAR-T and utilize AI technology for optimization. Through these comprehensive strategies, their safety is expected to improve significantly, accelerating the clinical translation process.

### 7.5 Further enhancing the therapeutic efficacy of CAR-exosomes

CAR-Exosomes therapy for solid tumors faces three major challenges: a limited selection of targets, the need for improved delivery efficiency, and the limitations of single-agent therapy. To address these bottlenecks, recent research focuses on three key areas. First, developing more targets for solid tumors is crucial. Given the heterogeneity of tumor cells, the development of CAR-Exosomes targeting multiple targets should also be explored, mirroring the success of dual-target CAR-T cell therapies (e.g., CD19/CD22) in refractory lymphomas (88). The future should see the development of multi-target CAR-Exosomes products. Second, integrating precision delivery systems to further enhance anti-tumor efficacy is essential. The application of inhaled PTX-loaded exosomes in lung tumors has significantly boosted drug concentrations (15). CAR-T-exosomes loaded with paclitaxel and fused with lung-targeting liposomes achieve drug enrichment in the lungs, enabling effective treatment of lung tumors and simultaneously reducing systemic toxicity (16). Third, the combination of CAR-Exosomes with multiple therapies may improve their therapeutic effect on tumors. For example, exosomes derived from γδ-T cells combined with radiotherapy can overcome radio-resistance in nasopharyngeal carcinoma cells significantly enhancing their therapeutic effect on nasopharyngeal carcinoma *in vitro* and *in vivo* (117). Furthermore, considering the potential of traditional Chinese medicine nanoparticles in immunotherapy (118), future research could explore the combined application of Chinese medicine nanoparticles with CAR-Exosomes. Additionally, multifunctional composite selenium nanoparticles have been proven to stimulate and enhance immune cell activity, particularly in antitumor treatments (119). These nanoparticles may be combined with CAR-exosome therapy. Therefore, this avenue warrants further attention, and efforts could be made to investigate the combined use of multifunctional composite selenium nanoparticles and CAR-Exosomes to achieve enhanced anti-tumor efficacy.

The rapid development of AI technology is becoming the core driver for strategies. In target discovery and screening: leveraging machine learning and deep learning algorithms, integrating multi-omics big data such as tumor genomics, transcriptomics, and proteomics, to predict and screen for highly expressed, highly immunogenic tumor-specific antigens. For instance, AI deep learning models have successfully identified the myeloid lineage markers CD86 and CSF1R as potential therapeutic targets for acute myeloid leukemia (120). Moreover, AI-driven simulation techniques, such as molecular dynamics simulations, can be used to optimize CAR-exosomes delivery strategies. For example, dynamic simulations of modified exosomes, predicting modification effects and influencing factors, can be performed to improve the targeting and stability of CAR-Exosomes within the tumor microenvironment. In terms of predicting efficacy and personalizing treatment, AI also holds significant promise. For example, studies have utilized CAR-T treatment datasets to train machine learning models, aiming to identify potential biomarkers of clinical outcomes. These efforts have successfully identified circulating lymphocyte subsets associated with clinical responses (121–123). In the future, AI technology can be used to integrate patient clinical data, imaging data, and gene expression profiles to build predictive models, thereby predicting the efficacy of CAR-Exosomes and providing a scientific basis for the development of personalized treatment plans.

### 7.6 Prolong half-life of exosomes

Current research generally indicates that naked, un-engineered natural exosomes exhibit a relatively short half-life in the bloodstream. Specifically, after intravenous injection, their half-life is typically only 2 to 7 minutes. This results in their very brief presence in the body, often becoming undetectable within minutes to a few hours (124–127). This swift clearance is primarily attributed to the rapid recognition and phagocytosis by the host immune system, particularly the robust mononuclear phagocyte system (MPS/RES) found in the liver and spleen. Recently, researchers have explored various strategies to extend the half-life of exosomes. CD47 modification, by engineering exosomes to overexpress or incorporate this ‘Don’t Eat Me’ signal, allows them to bind to phagocytic cell surface SIRPα receptors, thereby inhibiting macrophage uptake and significantly prolonging plasma half-life for extended therapeutic action (128). Similarly, the conjugation of polyethylene glycol (PEG) molecules to exosome surfaces, creates a hydrophilic barrier that effectively lowers immunogenicity and non-specific binding, reducing MPS/RES clearance and, as demonstrated by some studies, extending half-life to approximately 10 hours (129). Beyond PEGylation, Polyoxazolines modification has also emerged as a promising option for stabilizing plasma exosomes, with research showing it can extend exosomes half-life six-fold within 6 hours post-injection (130), highlighting its immense potential. In addition, genetically modifying source cells to secrete exosomes pre-loaded with desirable membrane proteins (e.g., enhanced anti-phagocytic signals) may confer intrinsic stability to derived exosomes, which needs to be further explored.

AI holds immense potential for extending exosome half-life in the future. AI can simultaneously consider multiple variables such as PEG molecular weight, density, and linking chemistry, CD47 expression levels, exosome surface charge, and more. Through complex algorithms, it can identify the optimal combination to achieve the longest half-life with minimal toxicity. AI can also analyze vast amounts of *in vivo* pharmacokinetic data, biodistribution data, and immune response data to identify and quantify the contribution of different clearance pathways (e.g., MPS clearance by the liver and spleen, renal filtration) to exosome elimination. This helps in discovering key molecular features that lead to rapid clearance, thereby providing clear engineering strategies to circumvent these clearance pathways in the future.

### 7.7 Exosome banking

To facilitate the therapeutic application of exosomes, the rapid development of exosome banking is crucial. The key to addressing this challenge lies in establishing and promoting a standardized process encompassing separation, purification, storage, and quality control, with a particular emphasis on maintaining exosome biological activity and integrity. Future strategies should focus on the following: First, integrating and optimizing multi-step combined separation strategies. Recognizing the limitations of single-method approaches, a multi-step approach can overcome drawbacks and enhance target purity and activity. For example, ultrafiltration or differential centrifugation can be used for initial concentration, followed by sophisticated purification techniques such as flow cytometry. Second, establishing a universally applicable standardized process. This involves developing standard operating procedures for sample collection, processing, separation, purification, quantification, QC, and storage, defining the optimal applicability of different methods and establishing evaluation standards for exosome purity, integrity, activity, and function. Third, on-going development and validation of novel and efficient technologies. This includes developing more rapid, gentle, high-throughput, highly specific techniques that can maintain exosome integrity and function, including but not limited to novel strategies for flow cytometry-based sorting, as well as AI and machine learning-driven sorting systems. Fourth, implementing rigorous multi-modal quality control. This requires the comprehensive application of techniques such as nanoparticle tracking analysis, transmission electron microscopy for morphological evaluation, exosome marker protein analysis by Western blotting, and RNA and lipidomic analysis, to ensure batch-to-batch consistency and biological activity of exosomes entering the bank. Fifth, further optimizing and developing innovative exosome storage strategies. This requires, in addition to existing cryopreservation methods, actively exploring new cryoprotective agents, optimization of lyophilization techniques, and stable storage strategies involving biomaterials. Furthermore, it’s crucial to conduct in-depth research to systematically optimize storage conditions and freeze-thaw limitations for different types of exosomes, and validate safer and more efficient cryoprotective agents.

In the future, we believe that AI can play a crucial role in resolving the challenges of exosome banking. AI can optimize separation processes through machine learning, automated systems, and the development of novel sorting technologies. It can utilize computer vision and machine learning to enhance image analysis, predict exosome bioactivity, and analyze batch-to-batch variations. Furthermore, AI can accelerate cryoprotectant screening and optimize storage conditions. Therefore, AI will be a significant driver in accelerating exosome banking construction and fostering the clinical application of exosome therapies.

## 8 Conclusions

In summary, CAR-Exosomes, as an emerging cancer therapy tool following CAR-T, CAR-NK, and CAR-M cell therapies, not only inherit the targeting and killing abilities of their predecessors but also integrate the nanoscale advantages of exosomes, providing excellent tissue penetration, enabling effective infiltration into the solid TME. This characteristic grants them unique potential in treating solid tumors, and they have already demonstrated, in initial studies, the absence of significant CRS or other severe toxic side effects. Despite facing clinical translation challenges, the CAR-Exosomes therapy holds great promise. Furthermore, with the multi-dimensional, high-potential solutions we have presented above, these challenges are expected to be mitigated in the future. Even more exciting is that the rapid development of AI technology provides unprecedented powerful driving force for the progress of CAR-Exosomes therapy. AI will empower various aspects of the CAR-Exosomes therapy, including: optimizing the production process, improving storage stability, deeply analyzing the mechanism of action, refining safety assessments, achieving personalized efficacy prediction; optimizing therapeutic efficacy, prolonging half-life and establishing exosome banking etc. We believe that with the continuous integration of cutting-edge technologies such as AI, CAR-Exosomes therapy will achieve breakthroughs in conquering solid tumors, bringing more precise, effective, and safer treatment options for cancer patients, and ushering in a new era of cancer treatment.
